# Digital signatures for early traumatic brain injury outcome prediction in the intensive care unit

**DOI:** 10.1038/s41598-021-99397-4

**Published:** 2021-10-07

**Authors:** Anil K. Palepu, Aditya Murali, Jenna L. Ballard, Robert Li, Samiksha Ramesh, Hieu Nguyen, Hanbiehn Kim, Sridevi Sarma, Jose I. Suarez, Robert D. Stevens

**Affiliations:** 1 Department of Biomedical Engineering, Whiting School of Engineering, Johns Hopkins University, Baltimore, MD USA; 2 Present Address: Department of Anesthesiology and Critical Care Medicine, Johns Hopkins University School of Medicine, Baltimore, MD USA; 3 Department of Neurology, Johns Hopkins University School of Medicine, Baltimore, MD USA; 4 Department of Neurosurgery, Johns Hopkins University School of Medicine, Baltimore, MD USA; 5 Division of Neuroscience Critical Care, Johns Hopkins University School of Medicine, 600 N. Wolfe St, Phipps Suite 455, Baltimore, MD 21287 USA

**Keywords:** Machine learning, Predictive medicine, Brain injuries

## Abstract

Traumatic brain injury (TBI) is a leading neurological cause of death and disability across the world. Early characterization of TBI severity could provide a window for therapeutic intervention and contribute to improved outcome. We hypothesized that granular electronic health record data available in the first 24 h following admission to the intensive care unit (ICU) can be used to differentiate outcomes at discharge. Working from two ICU datasets we focused on patients with a primary admission diagnosis of TBI whose length of stay in ICU was ≥ 24 h (N = 1689 and 127). Features derived from clinical, laboratory, medication, and physiological time series data in the first 24 h after ICU admission were used to train elastic-net regularized Generalized Linear Models for the prediction of mortality and neurological function at ICU discharge. Model discrimination, determined by area under the receiver operating characteristic curve (AUC) analysis, was 0.903 and 0.874 for mortality and neurological function, respectively. Model performance was successfully validated in an external dataset (AUC 0.958 and 0.878 for mortality and neurological function, respectively). These results demonstrate that computational analysis of data routinely collected in the first 24 h after admission accurately and reliably predict discharge outcomes in ICU stratum TBI patients.

## Introduction

Traumatic brain injury (TBI) is a leading cause of death and disability, with more than 50 million cases reported annually worldwide^1^. Among TBI patients admitted to the Intensive Care Unit (ICU), an estimated two thirds die or have neurological disability at 6 months^2^. To develop therapeutic interventions that improve outcomes, effective methods are needed to characterize TBI severity and predict clinical outcomes in the acute phase^3,4^. Established TBI prognostic scores such as the Corticosteroid Randomization After Significant Head Injury (CRASH) and International Mission for Prognosis and Analysis of Clinical Trials in TBI (IMPACT) combine clinical features (core models) or clinical features combined with head CT and laboratory or physiological variables (extended models) in multivariable logistic regression models^5,6^. These models have been tested and validated extensively and discriminate moderately well with areas under the receiver operator characteristic curve (AUC) of 0.82 and 0.79 across studies for CRASH and IMPACT respectively^7^).

One potential limitation of existing models is that they do not capture some important predictive features in this population. A more granular analysis of physiological signals (e.g. curve shape, local averages) may reveal important information about a patient’s clinical trajectory. Moreover, recent research indicates that prediction of clinical outcomes and physiological state transitions might be enhanced by training machine learning classifiers because they can effectively model granular relationships in high-dimensional spaces^8,9^.

Here, we explored electronic health record data to test the hypothesis that early data signatures can differentiate short-term clinical trajectories of TBI patients admitted to the ICU. We demonstrate that information available in the first 24 h of intensive care is predictive of mortality and neurological function at ICU discharge, and that machine learning models can accurately model this relationship. We found that model performance was robust and validated effectively in an independent external population.

## Results

### Study population

**Figure 1:**
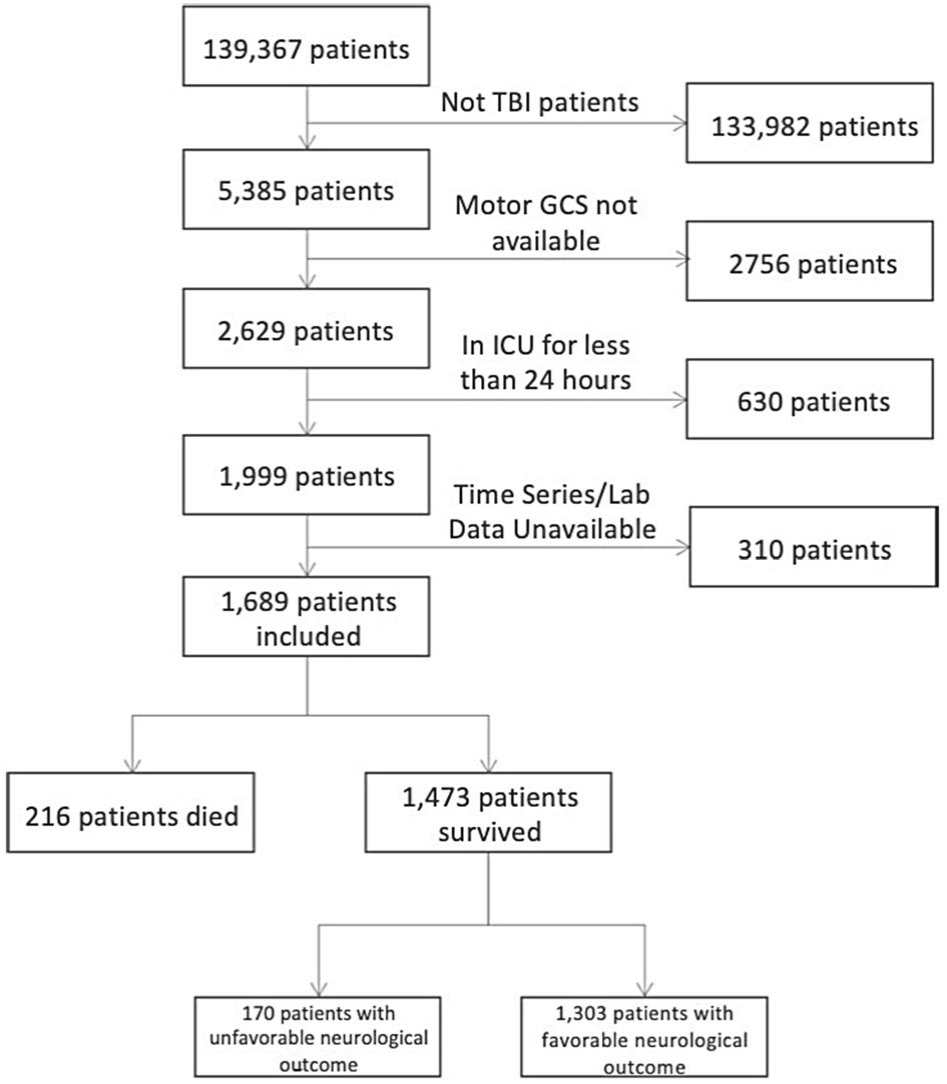
Study flow diagram. Shown are the patient inclusion and exclusion process and the outcomes recorded at ICU discharge.

A flow diagram illustrating patient selection and outcomes is provided in Fig. 1 and characteristics of the included patients are in Table 1. We identified ‘TBI Patients’ (population of 5,385 in eICU) as ICU admissions associated with the keyword ‘trauma—CNS’ contained in the ‘diagnosisstring’ variable in the eICU diagnosis table.

**Table 1:** Characteristics of patients in eICU multi-center database used for training machine learning models.

Variable	Survival status at ICU discharge		Neurological function at ICU discharge	
	Dead (N = 216)	Alive (N = 1473)	Unfavorable* (N = 170)	Favorable* (N = 1303)
Age (years)	61.6 ± 22.4	60.9 ± 21.6	64.3 ± 22.1	60.5 ± 21.5
Male Gender %	74.5% (161)	59.2% (872)	60% (102)	59.1% (770)
ICU stay length (days ± SD)	6.8 ± 7.7	8.8 ± 8.3	13.6 ± 11.3	8.1 ± 7.6
Mean ±SD admission GCS	9.7 ± 4.3	13.2 ± 2.69	7.8 ± 4.0	12.1 ± 4.0
Mean ± SD APACHE IV Score	89.9 ± 28.6	52.0 ± 21.6	70.1 ± 23.3	49.4 ± 20.5
N (%) receiving mechanical ventilation	102 (47.2%)	222 (15.1%)	84 (49.4%)	268 (20.6%)

Unfavorable neurological outcome refers to patients with Glasgow Coma Scale motor subscore of < 6 at the time of discharge. Favorable neurological outcome refers to patients with Glasgow Coma Scale motor subscore of 6 at discharge.

*GCS* Glasgow Coma Scale, *APACHE* acute physiology and chronic health evaluation.

### Model performance and external validation

**Figure 2:**
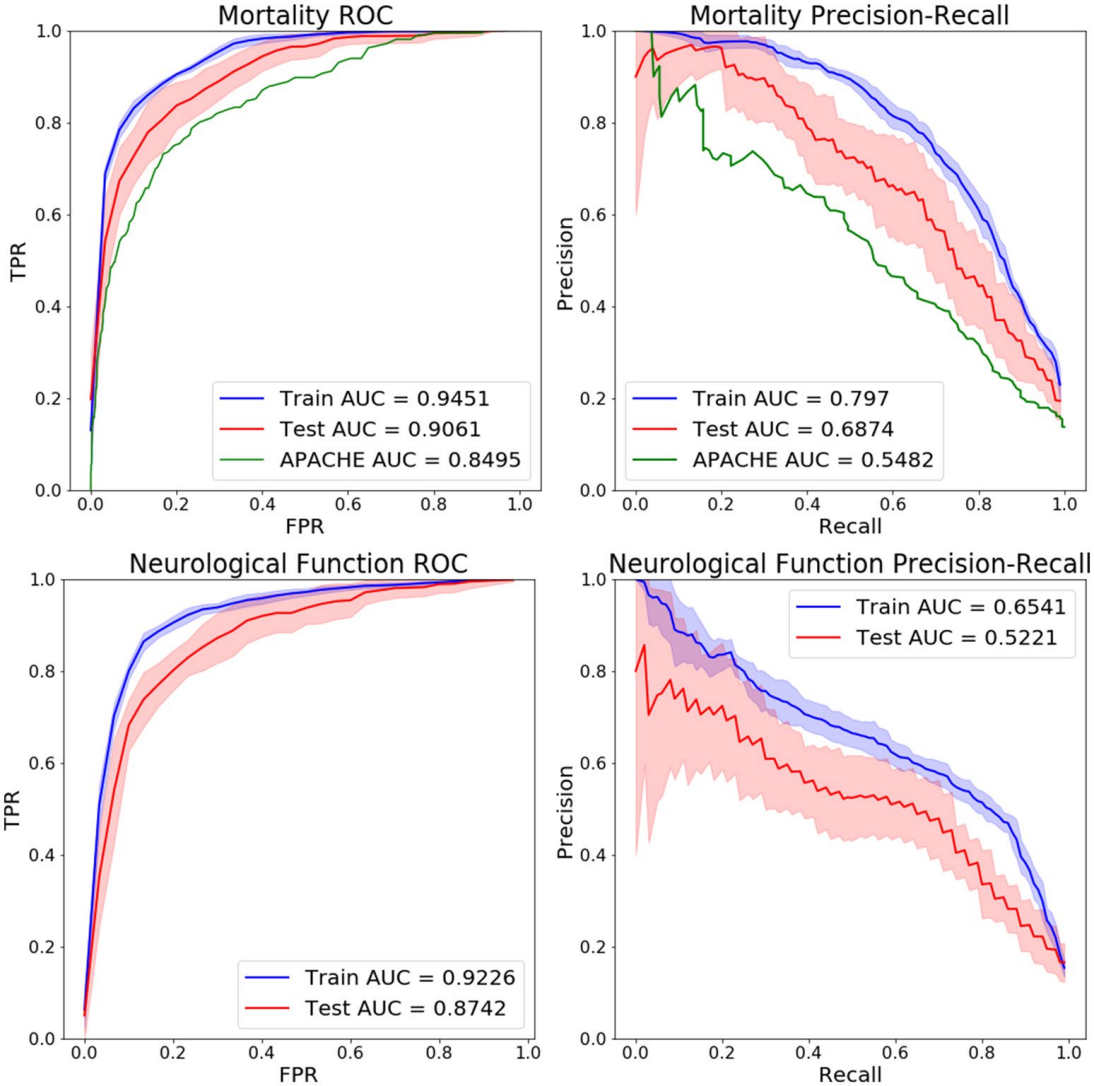
Receiver operating characteristic and precision recall curves. The blue lines and red lines correspond to results on the training sets and testing sets respectively, with the shaded error corresponding to the standard deviation across the 20 bootstrapped train-test splits. The green lines correspond to the existing APACHE-IV mortality prediction model evaluated on the eICU patients. ROC, Receiver operating characteristic. *AUC* area under the curve, *TPR* true positive rate, *FPR* false positive rate, *GCS* Glasgow Coma Scale.

**Figure 3:**
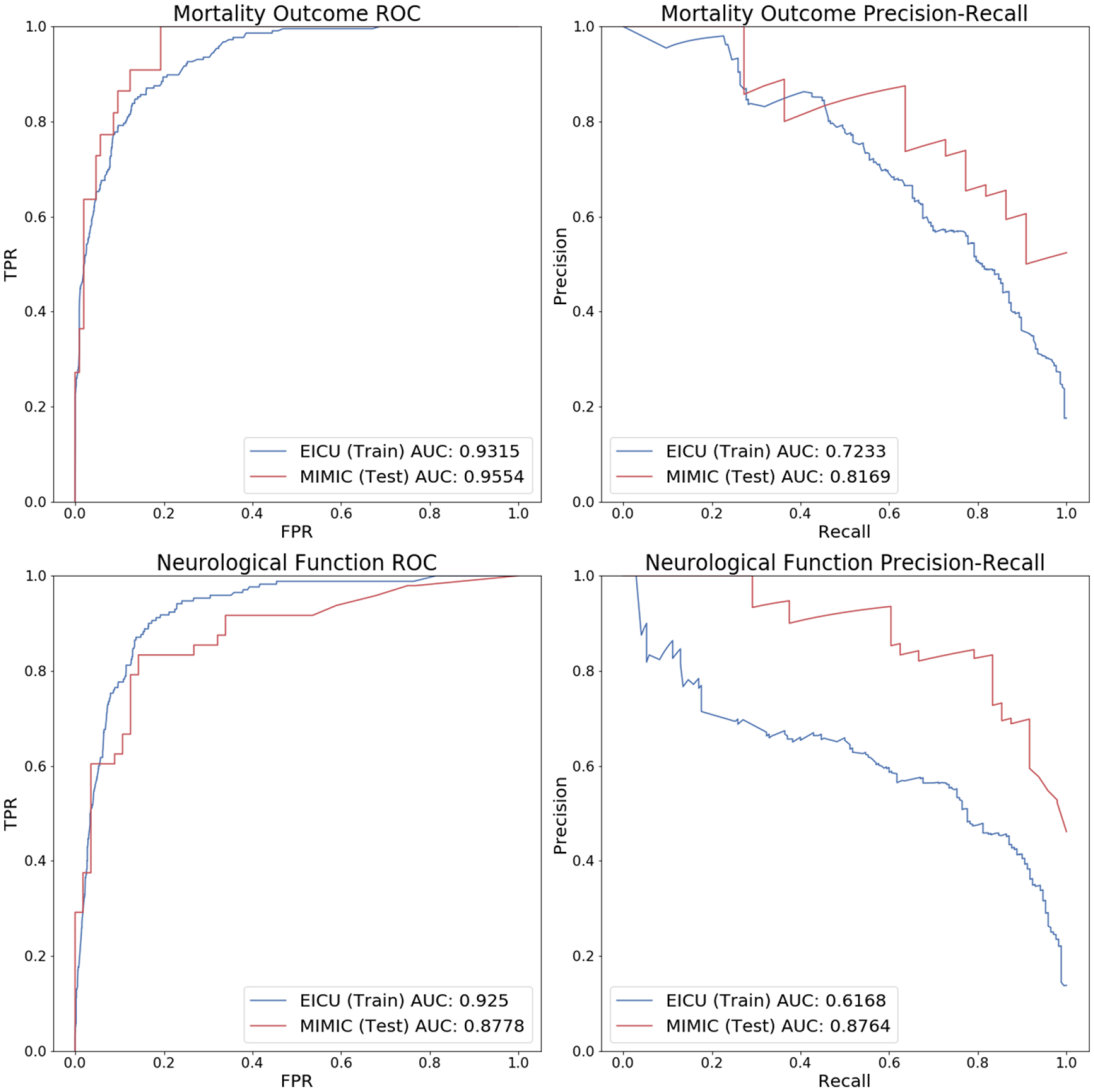
External validation. The blue line corresponds to results on the entire eICU data set, which is used to train the model, while the red line corresponds to results on the MIMIC-III patients, which are not used by the model until test-time. ROC, Receiver operating characteristic. *AUC* area under the curve, *TPR* true positive rate, *FPR* false positive rate, *GCS* Glasgow Coma Scale.

Model performance characteristics are presented in Figs. 2 and 3 and in Table 2. The elastic-net regularized GLM trained with the eICU-derived data accurately predicted mortality and neurological function as shown in Fig. 2. Results of model external validation in the MIMIC-III tertiary care hospital TBI population are shown in Fig. 3. There was marginal loss of discrimination and precision-recall, but overall model performance was maintained in the independent external dataset. This reiterates the efficacy of the weighted loss approach to mitigate class imbalance, as the MIMIC-III database was far more balanced than the eICU database (see Table 1).

**Table 2:** Model performance metrics.

Metric	Mortality prediction			Neurological outcome prediction		
	Training (eICU)	Validation (eICU)	Validation (MIMIC-III)	Training (eICU)	Validation (eICU)	Validation (MIMIC-III)
Sensitivity	0.840	0.786	0.818	0.866	0.791	0.813
Specificity	0.897	0.870	0.923	0.869	0.816	0.857
PPV	0.556	0.495	0.692	0.465	0.392	0.857
NPV	0.975	0.965	0.960	0.980	0.969	0.842
Discrimination (AUC)	0.945	0.906	0.958	0.923	0.874	0.878
Precision/Recall (AUPRC)	0.797	0.688	0.843	0.654	0.522	0.879
Precision/Recall (F1)	0.666	0.598	0.750	0.605	0.510	0.821
Calibration Index	0.104	0.125	0.498	0.210	0.410	0.000
Brier Score	0.051	0.065	0.065	0.061	0.074	0.265

eICU Training refers to the mean measurements across 20 randomly sampled subsets of the eICU data. eICU Validation refers to the mean measurements for the validation subsets corresponding to the 20 train samples. MIMIC Validation refers to the measurements collected by evaluating models trained on all of the eICU data on the MIMIC-III database. The model was evaluated with a positive prediction referring to unfavorable outcome (death labeled as 1, discharge mGCS ¡6 labeled as 1). The relatively low PPV for the two eICU datasets (training and validation) in the model partially result from a high class imbalance resulting in few positive examples (216, or 12.8% for mortality, 170, or 11.5% for neurological outcome). The MIMIC data had markedly lower class imbalance, leading to a higher PPV.

*NPV* negative predictive value, *PPV* positive predictive value; *AUC* area under the receiver operating characteristic curve, *AUPRC* area under the precision recall curve, *F1* F1 score or the harmonic mean of precision and recall, *eICU* eICU Clinical Research Database, *MIMIC* Medical Information Mart for Intensive Care-III database.

### Feature analysis

**Figure 4:**
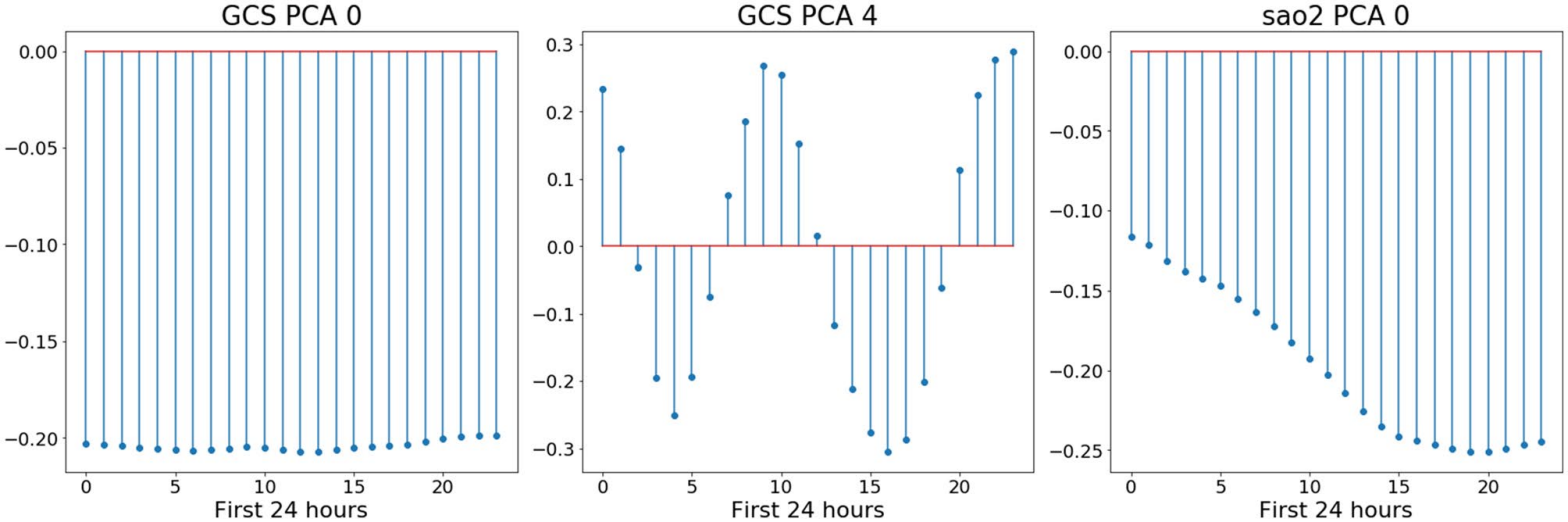
Principal component analysis. The plots indicate how three highly predictive principal component analysis terms (GCS component 0, GCS component 4, SaO_2_ component 0) are calculated. Prior to PCA, we calculate the mean of each vital measurement for each hour of a patient’s first 24 h in ICU, resulting in vector with 24 values. These values are then normalized to have 0 mean. Each graph shows the weight applied to each of the 24 hourly values of the component to compute the PCA component value. For example, GCS component 0 is computed by multiplying the normalized motor GCS at each hour by ~ − 0.2. The component has a high value if the patient has a low motor GCS throughout the first 24 h of their stay (high value of GCS component 0 is associated with poor neurological outcome and increased mortality). SaO_2_ component 0 has a high value if SaO_2_ is initially slightly below average (taken across all TBI patients) and continues to decrease, where high values of the component are associated with increased mortality. Finally, GCS component 4 has a high value if motor GCS oscillates over the first 24 h, starting above average. High values of this component (fluctuating level of consciousness) are associated with favorable neurological outcome.

A list of all features used in training model is provided in Supplementary Figure S1. The twenty features whose coefficients had the greatest weight in the final prediction of each outcome are shown in Supplementary Tables S2 and S3. These features included indicator variables, such as medication and nurse charting data, as well as PCA components of physiologic time-series signals such as pulse oximetry and heart rate (Fig. 4 and Supplementary Figure S1). As illustrated in Fig. 4, PCA revealed novel patterns in the time-series data that are highly predictive. For example, a motor GCS that oscillated with time was correlated with favorable neurological recovery, while a low and declining oxygen saturation was associated with low probability of survival.

## Discussion

These results demonstrate that computational models leveraging clinical and physiological data from the first 24 h of intensive care accurately predict short-term mortality and neurological function in patients admitted to the ICU for management of TBI. These prediction models were trained with physiological time series as well as laboratory and medication data, all of which are routinely collected in the ICU but for the most part are not used in current prognostic scores such as IMPACT, CRASH or APACHE. The performance of our models suggests that these additional features contain predictive information not available in the older prognostic scores. Furthermore, these models were robust in cross-validation and external validation.

Interpretability was a key priority in model design. The majority of predictive features selected by the models as having a significant impact on outcome were clinically and/or biologically plausible. For example, the first PCA component (capturing highest percentage of data variance of the mGCS), which encodes consistently low mGCS during the first 24 h after ICU admission, was the most predictive feature of unfavorable outcome for both mortality and end-of-stay neurological function (Fig. 4). In addition, previously unreported features were found to be associated with a higher likelihood of death or unfavorable neurological function, including several commonly used sedative and opioid medications, suggesting factors warranting further investigation.

Supplementary Tables S2 and S3 show the top 20 features ranked by predictive value for respectively the neurological function and mortality outcomes. Some of these features confirm results of prior studies. Associations were found, for example, between worse outcome at discharge and higher blood glucose levels or use of vasopressor infusions. Stress hyperglycemia and hypotension are known prognosticators which have been identified in previous TBI models (4,5). The apparent relationship between outcome and other variables (e.g. administration of morphine, odansetron, serum phosphate levels, basophil counts) will need to be examined in prospective multivariable models with appropriate adjustment for confounding.

While high interpretability was a key priority, we also wanted to ensure that we did not sacrifice model performance in the process. Our predictive models were able to achieve both of these goals, and moving forward, we plan to integrate other variables that might increase predictive power, such as features extracted from neuroimaging and neurophysiological studies. We plan also to prospectively test these models and develop prediction tools that could be integrated into the clinician’s workflow and enable accurate and timely decision-making in the ICU, possibly in quasi-real time. Studies could measure differences in accuracy between human and ML prediction, as well as how these assessments differentially evolve over the course of a patient’s trajectory. A major goal is to refine these ML models to a level where they can accurately predict outcomes at the subgroup or individual level, providing the basis for precise and personalized TBI care and motivating efforts to meaningfully integrate these predictions into clinical workflows. A related future line of research is to build on these ML models to predict short-term clinical and/or physiologic changes (patient state in the next 2–4 h) in severe TBI patients, and to predict responses to therapeutic interventions. Such research might be strengthened by additional features such as neuroimaging and higher frequency physiology or neurophysiological data to achieve meaningful results. Prospective model testing and validation would need to carefully determine if clinician-based treatment factors (e.g. medications, surgical interventions) can be retained in the model or should be excluded.

### Limitations

Several limitations of this work should be noted. The eICU and MIMIC-III registries are rich sources of data on patients admitted to ICUs, yet they lack elements which might be critical for clinically meaningful prognostication in TBI, including certain clinical features (such as pupillary reactivity), as well as information from brain imaging, which are important predictive variables in moderate and severe TBI patients^7^. In consequence, direct comparisons between our model and the IMPACT and CRASH models were not possible. Also of note, the clinical outcomes available in eICU are limited to survival status without provision of any validated post-TBI functional outcome scale such as the Glasgow Outcome Scale or Glasgow Outcome Scale Extended, and without any data beyond the time of ICU discharge. We defined an operational “neurological function” outcome based on a mGCS dichotomized at 6 (favorable) vs 1–5 (unfavorable); we believe that this is a clinically meaningful cutoff, since it separates patients who are awake and able to follow commands from others; however, this was a pragmatic approach which does not capture nuances of the functional and cognitive states observed in patients recovering from moderate and severe TBI. Due to the reliance on GCS scores as both an input feature and as an outcome, we had to exclude a large number of patients from the original TBI patient population in the eICU database (51.2%) in whom these measurements were missing. While this poses the risk of bias, external validation on the MIMIC-III database suggests that these models could be generalizable to a significant proportion of TBI patients admitted to ICU. Additional limitations are that we did not evaluate for multitrauma as a predictive feature, or for interhospital referrals, as detailed information was not available on these factors. Lastly, this study was a retrospective analysis conducted on prospectively collected data, and therefore carries all the inherent biases of retrospective studies.

## Conclusions

In ICU stratum TBI patients, parsimonious computational models trained with data available in the first 24 h after admission accurately predict ICU discharge mortality and neurological responsiveness. Confidence in the models was strengthened by successful external validation in a large independent dataset. The models were interpretable and suggested predictive features that warrant further investigation. They indicate that routinely collected electronic health record data contain early signatures which may help differentiate between clinical trajectories in intensive care. Timely characterization of severity and clinical trajectories could open a window for targeted interventions to ameliorate outcomes in patients with moderate and severe TBI. Research is needed to explore the sources of this early signal, and to determine the efficacy of such prognostic paradigms in a prospective setting.

## Methods

### Data sources

Data were extracted from the Philips eICU Collaborative Research Database (eICU)^10^ and Medical Information Mart for Intensive Care–III (MIMIC-III)^11^, two datasets which are in the public domain and were established from the deidentified electronic health records of ICU patients. eICU is a multi-center dataset which comprises 200,859 patient unit encounters for 139,367 unique patients admitted between 2014 and 2015. MIMIC-III database contains 55,000 hospital admissions (38,597 patients) admitted to ICUs of a single tertiary care medical center between 2001 and 2012.

### Patient inclusion criteria

The patient selection process is illustrated in Fig. 1. Patients were included if they were adults (> 18 years) admitted to the ICU and remained in intensive care > 24 h. We assumed that patients whose length of stay in ICU was greater than one day would have more severe injury and would have complete physiologic and laboratory data. We excluded patients with less than one measurement of each of the selected physiology variables (heart rate, respiratory rate, oxygen saturation) and less than 90% of laboratory measurements required in the model.

### Feature extraction

Only demographic, clinical, physiology, and intervention data that were available in the first 24 h of ICU stay were used in analysis. We implemented several different feature extraction strategies for physiology, laboratory, intervention, and demographic data. Heart rate, respiratory rate, oxygen saturation, temperature, verbal GCS, motor GCS, and eyes GCS, were treated as seven different continuous time series. Because measurement frequency varied by patient and component, we computed a 24-value summary vector for each measurement and each patient containing the mean value of that measurement during each hour of their stay (from hours 1 to 24). We conducted Principal Components Analysis (PCA) on these summary vectors, extracting the top 5 principal components (directions of highest variance in the data) for each measurement. The number of PCA components was selected with the intent of capturing 90% of the overall variance in the original data. Of note, the identification of the optimal PCA transformation matrix was conducted using only the training data, and this transformation matrix was applied to the evaluation data at test-time. For the 43 lab measurements that were present in at least 90% of our patients, the mean value for each lab was computed across the first 24 h. Meanwhile, we represented interventions (administered medications, medication infusions and mechanical ventilation) data using a vector of indicator variables describing the entirety of the first 24 h of the patient’s stay. A feature would be given a value of 1 if a patient received that particular treatment one or more times during the first day of their ICU stay, and a value of 0 otherwise. For the demographic data, dummy encoding was implemented to transform the categorical variables (ethnicity and gender), into dichotomized vectors. The remaining quantitative measurements (age, weight, height) were kept as numerical values.

### Outcome variables

The two primary outcome variables were death and neurological function at discharge from the ICU. Neurological function was defined operationally using the motor subscore of GCS (mGCS) recorded at ICU discharge or less than 24 h before ICU discharge; this endpoint was selected since validated TBI outcome measures are not recorded in eICU or MIMIC-III. Favorable neurological function was defined as a mGCS of 6, while unfavorable neurological function was defined as a mGCS of < 6. This was felt to be a clinically meaningful categorization since it differentiates patients who can follow commands from those who cannot.

### Class imbalance

A total of 1,689 TBI patients in eICU were selected for model training and validation. This final patient population was characterized by a significant class imbalance: 87% of patients survived their ICU stay. A similar pattern was observed in neurological outcome, with 88% of surviving patients having a final motor GCS of 6 (favorable). To mitigate the class imbalance, a weighted loss function was implemented during model training as follows:

 where *p*(class(*i*)) represents the proportion of the class of example *i* in the training dataset.

A similar class imbalance was noted in the external MIMIC-III test database, but it was less pronounced: 104 out of the 127 TBI patients survived (82%), and among those who survived, 56 had a final motor GCS of 6 upon discharge (54%).

### Analysis and modeling

The eICU data was divided into training and testing subsets with a 70–30 split ratio. Stratified sampling was employed to ensure that these splits contained similar distributions of the outcome variables. Within the training set, fivefold cross-validation was used to optimize the model. We chose to use an elastic-net penalized generalized linear model^12^ and conducted a grid-search across a variety of penalty and L1 ratio values to optimize cross-validation score. Using the parameters that yielded the highest cross-validation score, we trained the corresponding model on all the training data, then evaluated its performance on the test set. This process was repeated with 20 bootstrapped samples, in order to assess the variability of these metrics and our model coefficients. This process was employed for both the survival and neurological function outcomes. We employed a generalized linear model (GLM) with a logit link function, as depicted in the following equation:

$$\log \left( {\frac{p}{1 - p}} \right) = \theta_{0} + \sum\limits_{i = 1}^{I} {\alpha_{i} f_{i} } + \sum\limits_{j = 1}^{J} {\beta_{j} g_{j} } + \sum\limits_{k = 1}^{K} {\gamma_{k} h_{k} }$$

For mortality prediction, the target variable $p$ represented the probability that a patient would die by the end of their ICU stay. For neurological function, the target variable $p$ represented the probability that a patient would be discharged from the ICU with a motor GCS of less than 6 (i.e., they would be unable to follow commands). The variables ${f}_{i}$ represent medication and infusion features with corresponding coefficients ${\alpha }_{i}$, the variables ${g}_{j}$ represent the physiology and GCS time series features (5 PCA components for each measurement) with corresponding coefficients ${\beta }_{j}$, and the variables ${h}_{k}$ represent the lab data features (vector of 43 measurements) with corresponding coefficients ${\gamma }_{k}$. All features were standardized to have zero mean and unit variance. The goal during training was to discover an assignment of the coefficients ${\alpha }_{i}$, ${\beta }_{j}$, ${\gamma }_{k}$ that minimized validation error across the 20 bootstraps.

### Evaluation of model performance

Prior to assessing model performance, models were calibrated by rescaling the predicted class probabilities during cross-validation to better reflect the true probability of each outcome^13^. Performance of the models were assessed with two principal measures: the Receiver-Operator Characteristic (ROC) and Precision-Recall (PR). The area under each of these curves (AUC) was used as the final performance metric for each model, with larger AUCs indicating more discriminatory power.

### Acute physiology and chronic health evaluation (APACHE) model

We were not able to directly test the CRASH and IMPACT models on our dataset, since key features required for these models (e.g. pupil reactivity, imaging results) are not available in eICU or MIMIC. As a reference model for comparison with our mortality prediction model, we used the APACHE IV score along with mortality labels to plot benchmark ROC and PR curves. The APACHE IV score is a multivariable model used to assess severity of illness and leverages features typically collected in the ICU. APACHE has been widely used as a benchmark in critically ill TBI patients^14–16^.

### External validation

External validation was conducted in the MIMIC-III database (n = 127 patients). The physiological time series features (heart rate, respiratory rate, pulse oximetry, temperature and GCS) are available in both the eICU and MIMIC-III databases. However, because some of the lab, medication, and infusion features differ between the two datasets, we limited our feature set to those that were available in both datasets. Then, for each outcome variable, we trained a new model using all of the TBI patients in eICU and evaluated the results on all TBI patients from the MIMIC-III database. These patients were chosen with the same inclusion/exclusion criteria as the patients from the eICU database.

### Ethics declarations

Research in this report was carried out on fully deidentified publicly available datasets made available via the Massachusetts Institute of Technology (MIT) PhysioNet repository (https://physionet.org/). Data in the MIMIC-III database have been deidentified, and the institutional review boards of MIT (number 0403000206) and Beth Israel Deaconess Medical Center (number 2001-P-001699/14) both approved the use of the database for research. Because the database does not contain protected health information, a waiver of the requirement for informed consent was included in the IRB approval. Data in eICU are also deidentified, and research using eICU data is exempt from institutional review board (IRB) approval due to the retrospective design, lack of direct patient intervention, and the security schema, for which the re-identification risk was certified as meeting safe harbor standards by an independent privacy expert (Privacert, Cambridge, MA, USA; Health Insurance Portability and Accountability Act Certification number 1031219–2). All methods were carried out in accordance with relevant guidelines and regulations.

## Supplementary Information

Supplementary Figure 1.

Supplementary Tables.

## Data Availability

Data analyzed in this study are publicly available from the eICU-CRD and MIMIC-III databases (https://physionet.org/).

## Code availability

All code used to extract, process, and analyze data will be made available on request.
