# Supplementary figures and images for: Digital signatures for early traumatic brain injury outcome prediction in the intensive care unit
Source: Sci Rep. 2021 Oct 7;11:19989. doi: 10.1038/s41598-021-99397-4 (PMC8497604; doi:10.1038/s41598-021-99397-4)

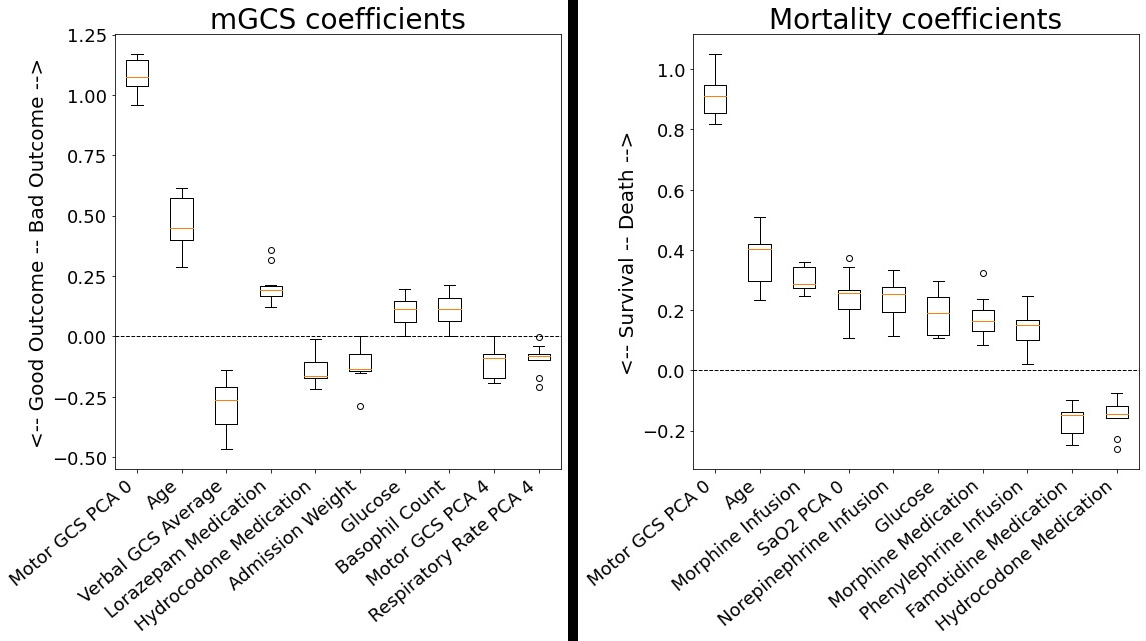

Supplement: Supplementary file 1 — Supplementary Figure 1. [file 41598_2021_99397_MOESM1_ESM.tif]
