# Supplementary material for: Digital signatures for early traumatic brain injury outcome prediction in the intensive care unit
Source: Sci Rep. 2021 Oct 7;11:19989. doi: 10.1038/s41598-021-99397-4 (PMC8497604; doi:10.1038/s41598-021-99397-4)
Supplement: Supplementary file 2 — Supplementary Tables. [file 41598_2021_99397_MOESM2_ESM.docx]

**Supplementary Materials**

# Digital Signatures for Early Traumatic Brain Injury Outcome Prediction in the Intensive Care Unit

**Contents**

Legend for Supplementary Figure 1

Supplementary Table 1

Supplementary Table 2

Supplementary Table 3

**Supplementary Figure 1: Feature analysis (Model Coefficients)**

The boxplots indicate top-ranked features that are most predictive of neurological outcome (left) and mortality (right) across 20 different train-test splits. Positive values indicate that a given feature is associated with unfavorable outcomes, while negative feature values are indicative of favorable outcome. The numbers for the time series features indicate a principal component derived from Principal Components Analysis (PCA).

| **Baseline Demographic Variables** | **Injury Severity Variables (Summarized with Average)** | **Physiological Variables (Summarized with PCA)** | **Medications**  **(0/1 Indicator Variable)** | **Drug Infusions**  **(0/1 Indicator Variable)** | **Laboratory Measurements (Summarized with Average Value)** |
| --- | --- | --- | --- | --- | --- |
| Age  Weight  Gender | Verbal GCS  Eyes GCS  Motor GCS 0  Motor GCS 1  Motor GCS 2  Motor GCS 3  Motor GCS 4  Temperature | Heart Rate 1  Heart Rate 2  Heart Rate 3  Heart Rate 4  Heart Rate 5  Respiratory Rate 1  Respiratory Rate 2  Respiratory Rate 3  Respiratory Rate 4  Respiratory Rate 5  Oxygen Saturation 1  Oxygen Saturation 2  Oxygen Saturation 3  Oxygen Saturation 4  Oxygen Saturation 5  Systolic Pressure (avg)  Diastolic Pressure (avg)  Blood Pressure (avg)  Intracranial Pressure (avg)  Central Venous Pressure (avg)  ICP (indicator)  CVP (indicator) | Potassium Chloride  Morphine  Hydrocodone-Acetaminophen  Metoprolol  Famotidine  Lorazepam  Glucagon  Ondansetron | Fentanyl  Insulin  Midazolam  Morphine  Nicardipine  Norepinephrine  Phenylephrine  Propofol  Red Blood Cell  Sodium  Vasopressin | Basophil Count  Eosinophil Count  Lymphocyte Count  Monocyte Count  Absolute Neutrophil Count  ALT (SGPT)  AST (SGOT)  Blood Urea Nitrogen  Base Excess  FiO_2_  HCO_3_  Hct  Hgb  MCH  MCHC  MCV  MPV  O_2_ Sat (%)  PT  PT - INR  PTT  Red Blood Cell Count  RDW  White Blood Cell Count  Albumin  Alkaline phos.  Anion gap  Bedside Glucose  Bicarbonate  Calcium  Chloride  Creatinine  Glucose  Magnesium  pH  PaCO_2_  PaO_2_  Phosphate  Platelet Count  Potassium  Sodium  Total Bilirubin  Total Protein |

**Supplementary Table 1: Directory of Model Training Features**

Shown is a directory of all features used for model training. The numbers for the periodic time series features indicate a principal component derived from Principal Components Analysis, where lower numbers indicate higher statistical significance. Unless otherwise stated, features in each column are all processed in the same manner. GCS, Glasgow Coma Scale. ICP, intracranial pressure. CVP, central venous pressure. ALT, alanine aminotransferase. AST, aspartate aminotransferase. Hct, hematocrit. Hgb, hemoglobin, MCH, mean corpuscular hemoglobin. MCHC, mean corpuscular hemoglobin concentration. MCV, mean corpuscular volume. MPV, mean platelet volume. PT, prothrombin time. INR, international normalized ratio. PTT, partial thromboplastin time. RDW, red cell distribution width.

**Supplementary Table 2: Top 20 Features for Neurological Function Prediction**

| **Ranking** | **Feature** | **Predictive Value**  **(Neurological Function)** |
| --- | --- | --- |
| 1 | Motor GCS PCA 1 | 1.08 |
| 2 | Age | 0.45 |
| 3 | Verbal GCS Average | -0.26 |
| 4 | Lorazepam Medication | 0.19 |
| 5 | Hydrocodone-acetaminophen Medication | -0.16 |
| 6 | Admission Weight | -0.14 |
| 7 | Glucose | 0.11 |
| 8 | Basophil Count | 0.11 |
| 9 | Motor GCS PCA 5 | -0.09 |
| 10 | Respiratory Rate PCA 5 | -0.08 |
| 11 | Ondansetron Medication | -0.08 |
| 12 | Magnesium | 0.07 |
| 13 | Fentanyl Infusion | -0.06 |
| 14 | Blood Pressure | 0.06 |
| 15 | Vasopressin Infusion | 0.05 |
| 16 | ICP (1 if recorded, 0 otherwise) | 0.05 |
| 17 | Glucagon Medication | 0.04 |
| 18 | Phosphate | 0.04 |
| 19 | Labetalol Medication | -0.04 |
| 20 | Sodium Infusion | 0.04 |

Shown are the top 20 most predictive features for mGCS. Predictive value for a feature was assessed by studying the GLM coefficient associated with the feature. Features are ranked by decreasing coefficient magnitude. Positive values indicate that larger values of the feature are associated with negative outcome, and vice-versa for negative values. Medication and infusion features were measured as 1 if administered at least once and 0 otherwise. PCA components were extracted for each continuous signal, and lower numbers indicate higher statistical significance. See Figure 4 for further analysis and discussion of these PCA features.

**Supplementary Table 3: Top 20 Features for Mortality Prediction**

| **Ranking** | **Feature** | **Predictive Value (Mortality)** |
| --- | --- | --- |
| 1 | Motor GCS PCA 0 | 0.91 |
| 2 | Age | 0.40 |
| 3 | Morphine Infusion | 0.29 |
| 4 | SaO_2_ PCA 0 | 0.26 |
| 5 | Norepinephrine Infusion | 0.25 |
| 6 | Glucose | 0.19 |
| 7 | Morphine Medication | 0.17 |
| 8 | Phenylephrine Infusion | 0.15 |
| 9 | Famotidine Medication | -0.15 |
| 10 | Hydrocodone-acetaminophen Medication | -0.15 |
| 11 | Heart Rate PCA 1 | 0.12 |
| 12 | Lorazepam Medication | 0.12 |
| 13 | Central venous pressure | 0.11 |
| 14 | Vasopressin Infusion | 0.10 |
| 15 | Blood Urea Nitrogen | 0.10 |
| 16 | White Blood Cell Count | 0.09 |
| 17 | Sodium | 0.09 |
| 18 | PaCO_2_ | 0.08 |
| 19 | Ondansetron Medication | -0.08 |
| 20 | Gender | 0.07 |

Shown are the top 20 most predictive features for mortality. Predictive value for a feature was assessed by studying the GLM coefficient associated with the feature. Features are ranked by decreasing coefficient magnitude. Positive values indicate that larger values of the feature are associated with negative outcome, and vice-versa for negative values. Medication and infusion features were measured as 1 if administered at least once and 0 otherwise. PCA components were extracted for each continuous signal, and lower numbers indicate higher statistical significance. See Figure 4 for further analysis and discussion of these PCA features.
